# Response of the multiple sclerosis community to COVID-19

**DOI:** 10.1177/1352458520948748

**Published:** 2020-09-14

**Authors:** Olga Ciccarelli, Jeffrey A Cohen, Alan Thompson

**Affiliations:** Queen Square Multiple Sclerosis Centre, Departmentof Neuroinflammation, UCL Queen Square Institute of Neurology, Faculty of Brain Sciences, University College London, London, UK/NIHR University College London Hospitals Biomedical Research Centre, London, UK; Queen Square Multiple Sclerosis Centre, Department of Neuroinflammation, UCL Queen Square Institute of Neurology, Faculty of Brain Sciences, University College London, London, UK; Mellen Center (J.A.C.), Cleveland Clinic, Cleveland, OH, USA

Soon after COVID-19 became a pandemic and the first countries introduced lockdown measures, multiple sclerosis (MS) experts did what they do best, which is working together and collegiately in order to (1) re-organise clinical MS services, (2) set up national and international COVID-19 and MS research networks, (3) recommend guidelines on MS management and (4) report national cohorts of COVID-19 in MS patients and cases with an unexpected COVID-19 course. In this issue of *Multiple Sclerosis Journal*, there are examples of each of these commendable and important activities, which we are going to highlight in turn. Remarkably, these activities were carried out over a very short period of time.

The COVID-19 pandemic has significantly impacted on the delivery of MS care by redeployment of staff to COVID-19 activities, allocation of beds to COVID-19 patients, suspension of routine blood tests and magnetic resonance imaging (MRI) scans in hospital, discontinuation of rehabilitation and the rapid adoption of telemedicine for non-urgent cases. In this issue of *Multiple Sclerosis Journal*, Sastre-Garriga et al.^1^ carefully describe all the adaptations implemented at the Multiple Sclerosis Centre of Catalonia (Cemcat) in Barcelona (Spain) in response to the pandemic, which will inspire other centres to introduce similar changes in order to provide the most effective care for MS patients. Some of their changes, such as home deliveries of infusions, phone and video consultations and online teaching material, are going to be long-lasting positive developments.

In the *Controversies* section in this issue of *Multiple Sclerosis Journal*, it is debated whether COVID-19 will change MS care forever. Meca-Lallana^2^ argues the yes position and suggests that as natalizumab-induced progressive multifocal encephalitis changed our clinical practice by introducing safety protocols based on testing of antibodies anti-JC virus, COVID-19 will lead to new safety protocols for drug administration based on determining SARS-CoV-2 RNA and serology. Long-term changes include the introduction of home drug delivery programmes and the increased use of digital tools to record patients’ self-assessments. Preziosa et al.^3^ take the contrary position that COVID-19 is not going to change MS care in the longer term and argue that the new strategies adopted to reduce the risk of COVID-19 in patients with MS have been taken on preventively and are not evidence based. Changes in patients’ monitoring, treatment and assessments are not going to be permanent, and the standard management of people with MS established in the pre-COVID-19 era is soon going to be resumed in order not to become a disadvantage for patients. Krieger^4^ identifies elements of truth in both arguments and summarises three levels of potential impact of the pandemic on MS: (1) the fundamentals of MS care, which have not been impacted; (2) our approach to clinical decision-making, including the use of more effective disease-modifying treatments (DMTs), for which evidence is missing and (3) the logistics we employ, such as the adoption of telehealth, which have been transformed significantly.

The rapid set up of a dedicated and coordinated network led by the MS International Federation (MSIF) and MS Data Alliance^5^ is described in this issue of the *Multiple Sclerosis Journal*. This network includes 23 international partners collecting a core dataset on COVID-19 in patients with MS, with the aim to understand the effect of DMTs on COVID-19 outcomes. Data are entered by clinicians and patients. In addition, all MS registries and cohorts are regularly sharing their COVID-19 core datasets into the central platform. Yam et al.^6^ recommend that pregnant MS women are included in all registries as a subgroup of interest, given their relatively small number. Importantly, this innovative and remarkable data sharing approach can be used in future as a template for different stakeholders to share data quickly and effectively and answer research questions which are non-COVID-19 related.

Individuals with MS, particularly those who are receiving high-efficacy treatments, have been postulated to be at increased risk of infection and mortality from COVID-19. This hypothesis had a significant impact on the treatment of patients. Despite the absence of secure evidence on the impact of DMTs on COVID-19, national and international treatment guidelines were recommended on the basis of expert consensus and the known biology of the mechanisms of action of DMTs.^1,7–9^ These guidelines recommended that delayed initiation or re-treatment should be considered when using high-efficacy and lymphocyte depleting drugs. The manuscripts published so far are reassuring.^10,11^ In this issue of *Multiple Sclerosis Journal*, Loonstra et al.^12^ describe the Dutch cohort of MS and COVID-19 patients. This cohort includes 86 MS patients, 43 of whom tested positive. A trend of a worse outcome in MS patients on DMTs in general is not detected, and a clear link between low lymphocyte count and severe disease is not observed. This agrees with three patients reported in the same issue of *Multiple Sclerosis Journal* who were treated with Rituximab, Cladribine and Alemtuzumab. All these patients recovered from COVID-19 despite lymphopenia, suggesting that SARS-CoV-2 clearance is possible without B cells, and T and B cells depleting therapies do not seem to prevent a fully clinical recovery. The first case report describes a patient on Rituximab who recovered from a moderate COVID-19 associated pneumonia, despite an undetectable B lymphocyte count.^13^ The second case is a patient with severe lymphopenia (expected to involve both B and T cells) who was treated with Cladribine 2 weeks before the onset of COVID-19 and recovered from a moderate course of COVID-19.^14^ The third patient developed COVID-19 symptoms 1 week after the second cycle of Alemtuzumab and recovered from a mild infection despite neutropenia and lymphopenia.^15^ In the Clinical Commentary which accompanies these three case reports, Brownlee^16^ suggests that the risk of disability worsening from delayed initiation or re-treatment with a high-efficacy treatment will outweigh the risks of severe COVID infection, and therefore, we are now ready to reinstitute the standard MS treatment protocols.

In the same issue of *Multiple Sclerosis Journal*, Yam et al.^6^ advise on the management of pregnancy in MS on the basis of data derived by the obstetric literature and previous coronavirus infections. The authors recommend that: pregnant women with MS maintain contact with obstetric services as appropriate to their particular circumstances and exercise social distancing after 28-weeks’ gestation; there are no contraindications to vaginal delivery unless there is maternal deterioration or foetal compromise; women who give birth during the COVID-19 pandemic should be encouraged to breastfeed where they wish to do so, with appropriate precautions in case of asymptomatic infection.

All the cohorts and cases published so far have highlighted the role of high disability, comorbidities, age and sex in increasing the risk of severe COVID-19 course.^11,12^ Moss et al.^17^ in the same issue of *Multiple Sclerosis Journal* describe the results of surveys sent to 10,816 MS patients at Cleveland Clinic, Johns Hopkins and Multiple Sclerosis Centre of Catalonia (Cemcat) in April/May 2020. Questionnaires were collected from 3028 (28%) patients and provided information about comorbidities, DMTs, exposures, COVID-19 testing/outcomes, health behaviours and disruptions to MS care. There were 77 (2.5%) cases of suspected or confirmed COVID-19. The results show that there is a good compliance with reported social-distancing measures and that individuals having to work on-site, with lower education level and residing in more socioeconomically disadvantaged areas are less likely to be following social-distancing guidelines. Therefore, effort should be made in order to direct educational interventions to younger people with lower socioeconomic status.

This issue of *Multiple Sclerosis Journal* provides excellent examples of the impressive response of MS experts and MS societies who worked together to tackle some of the most critical issues faced during the COVID-19 pandemic. This effort has to continue in order to understand the effects of DMTs on the immune response to SARS-CoV-2 and COVID-19 outcomes in MS patients across all levels of disability and different phenotypes. A successful strategy that needs to be embraced in order to achieve this goal is to calculate the overall incidence of COVID-19 positivity and outcome among MS patients by inputting secure numerators (number of new cases) and denominators (size of the population) for a specific period of time and collect information on clinical characteristics and disease outcomes.
